# The relative risk of immune checkpoint inhibitor pneumonitis in advanced non-small- cell lung cancer: Meta-analyses of controlled clinical trials

**DOI:** 10.1371/journal.pone.0301931

**Published:** 2024-04-29

**Authors:** Ying Kong, Liang Hong, Xiao-cheng Xu, Yun-feng Chen, Jia Xu

**Affiliations:** 1 Department of Oncology, The First People’s Hospital of Xiaoshan District, Xiaoshan Affiliated Hospital of Wenzhou Medical University, Hangzhou, Zhejiang, China; 2 Department of Radiology, The First People’s Hospital of Xiaoshan District, Xiaoshan Affiliated Hospital of Wenzhou Medical University, Hangzhou, Zhejiang, China; UT MD Anderson Cancer Center, UNITED STATES

## Abstract

**Objective:**

Immune checkpoint inhibitor pneumonitis (CIP) is a prevalent form of immunotherapy-induced pulmonary toxicity, ranking among the leading causes of mortality associated with immune checkpoint inhibitors (ICIs). Despite its significance, the risk stratification of CIP in advanced non-small cell lung cancer (NSCLC) remains uncertain. In this study, we conducted a comprehensive analysis, comparing various factors such as histological types, treatment regimens, PD-L1 expression levels, and EGFR/ALK negativity in advanced NSCLC. Our investigation extends to evaluating the relative risk of developing CIP based on previous treatment history. This analysis aims to provide valuable insights for the identification of specific patient subgroups at higher risk, facilitating more effective risk management and precision therapy approaches.

**Methods:**

PubMed, Embase, and Cochrane databases were systematically searched up to February 16, 2023. We conducted a screening of randomized controlled trials (RCTs) that compared ICI monotherapy or its combination with chemotherapy in advanced NSCLC. The trials were categorized based on histological type, treatment regimen, PD-L1 expression level, EGFR/ALK-negative status, and prior treatment history. Subsequently, the data were stratified into five subgroups, and the occurrences of all-grades (1–5) and high-grades (3–5) pneumonia events were extracted. Odds ratios (OR) and corresponding 95% confidence intervals (CI) were then calculated for further analysis.

**Results:**

Twenty-two RCTs, encompassing 13,725 patients with advanced NSCLC, were included in this analysis. Regardless of histology (OR = 2.47, 95% CI 1.41–4.33, P = 0.002; OR = 1.84, 95% CI 1.10–3.09, P = 0.02), treatment regimen (OR = 3.27, 95% CI 2.00–5.35, P < 0.00001; OR = 2.91, 95% CI 1.98–4.27, P < 0.00001), PD-L1 expression level (OR = 5.11, 95% CI 2.58–10.12, P < 0.00001; OR = 5.15, 95% CI 2.48–10.70, P < 0.0001), negative EGFR/ALK expression (OR = 4.32, 95% CI 2.22–8.41, P < 0.0001; OR = 3.6, 95% CI 1.56–8.28, P = 0.003), whether there is a history of treatment (OR = 3.27, 95% CI 2.00–5.35, P < 0.00001; OR = 2.74, 95% CI 1.75–4.29, P < 0.0001), ICI use was associated with a higher risk of all-grade (1–5) and high-grade (3–5) pneumonia compared to chemotherapy. Subgroup analysis revealed that the squamous group, the ICI vs. combination chemotherapy (CT) group, the PD-L1 > 50% group, and the previously untreated group had a higher risk of developing all-grade and grade 3–5 CIP (P < 0.05).

**Conclusions:**

In advanced NSCLC, ICI treatment was linked to an elevated risk of pneumonitis across all grades (1–5) as well as high-grade occurrences (3–5) compared to chemotherapy. Notably, individuals with squamous histology and high PD-L1 expression, along with those lacking a history of prior treatment, demonstrated a heightened susceptibility to developing immune-related pneumonitis of all grades (1–5) and high grades (3–5). These observations provide valuable insights for clinicians seeking to enhance the management of pulmonary toxicity associated with immunotherapy.

## Introduction

Worldwide, lung cancer stands as the leading cause of cancer-related deaths [1]. Over 80% of these cases are non-small cell lung cancer (NSCLC) [2], often diagnosed at an advanced or metastatic stage. While targeted therapies are available for molecularly defined advanced NSCLC patients, such as those with EGFR mutations or ALK rearrangements, a significant proportion of NSCLC cases lacking such genetic mutations renders these therapies ineffective. In recent years, the advent of immune checkpoint inhibitors (ICIs), including programmed cell death protein 1 (PD-1), programmed cell death ligand 1 (PD-L1), and cytotoxic T lymphocyte-associated antigen 4 (CTLA-4), has substantially improved the overall survival of NSCLC patients, establishing itself as the standard treatment paradigm [3].

The treatment goals for patients with advanced NSCLC aim to optimize survival, maintain quality of life, and minimize treatment-related side effects. With the increasing utilization of immune checkpoint inhibitors (ICIs) in advanced NSCLC, there is a growing focus on the adverse reactions associated with immunotherapy. Immune checkpoint inhibitor pneumonitis (CIP) is a prevalent form of immunotherapy-related pulmonary toxicity, which can range in severity, including instances that may be fatal [4,5]. CIP is commonly associated with PD-1 inhibitor therapy but can also manifest with PD-L1 or CTLA-4 inhibitor therapy [6].

Results from two meta-analyses have indicated that the overall incidence of CIP in lung cancer patients is higher than in patients with other types of tumors [5,7]. The incidence of fatal CIP is reported to be between 0.2% and 0.5% [8]. In non-small cell lung cancer treated with ICIs monotherapy, the overall incidence of CIP ranges from 3.1% to 4.1%, with the incidence of grade 3–5 CIP at 1.4%. PD-1 inhibitors exhibit a higher incidence of CIP (3.6% vs. 1.3%) and a greater frequency of severe CIP (1.1% vs. 0.4%) compared to PD-L1 inhibitors [7]. In contrast to ICIs monotherapy, immune combination therapy (involving immunotherapy combined with chemotherapy, double immunotherapy, immunotherapy combined with radiotherapy, or molecular targeted drugs) can elevate the risk of CIP [9]. A meta-analysis of ICIs combined with chemotherapy revealed a relative risk (RR) of 2.37 (95% CI 1.27–4.32, P = 0.007) for CIP with combined therapy, underscoring the increased risk associated with immune combination chemotherapy [10].

While numerous researchers have extensively examined the characteristics of CIP in NSCLC, further investigation into risk stratification is imperative. The pivotal factors influencing the selection of initial treatment options for advanced NSCLC encompass the histological type, distinguishing between squamous and non-squamous varieties, the presence of driver gene mutations, such as EGFR, ALK, and ROS1 mutations, and the expression of PD-L1. Despite these considerations, there has been a lack of meta-analyses or systematic reviews addressing risk stratification for the occurrence of CIP in advanced NSCLC, considering histology, biological characteristics, and clinical therapeutics.

In this study, we undertook the stratification of CIP risk within five subgroups of advanced NSCLC based on distinct histologies, treatment regimens, PD-L1 expression levels, EGFR/ALK negativity, and prior treatment history. Our objective was to explore the Odds Ratios (OR) associated with all-grade and grade 3–5 CIP in patients with advanced NSCLC undergoing treatment with PD-1/PDL-1 or CTLA-4 inhibitors. This analysis aims to assist clinicians in reinforcing the management of immunotherapy-related pulmonary toxicity and determining the necessity of preventive treatments for specific patient subgroups.

## Methods

### Search strategy

As of February 16, 2023, we have retrieved eligible randomized controlled trials (RCTs) from PubMed, Embase, and Cochrane. The keywords employed in the search included "immune checkpoint inhibitor," "advanced non-small cell lung cancer," "chemotherapy," "pneumonia," and "immune-related adverse events." Furthermore, we conducted searches for additional studies in the major proceedings of the American Society of Clinical Oncology (ASCO), the American Association for Cancer Research, the European Society for Medical Oncology (ESMO), and the World Congress on Lung Cancer (WCLC).

Our inclusion criteria were as follows: (1) Population: patients with pathologically confirmed stage IIIB or IV NSCLC; (2) Intervention: ICI monotherapy or combination with chemotherapy (CT); (3) Comparison: single-agent CT or combination CT; (4) Outcome: CIP of all grades, and grade 3–5, measured as an OR. The exclusion criteria were: (1) non-RCT; (2) lack of relevant data.

### Data extraction and quality assessment

Two authors (KY and HL) independently conducted reviews in accordance with the Preferred Reporting Items for Systematic Reviews and Meta-Analysis (PRISMA) guidelines. They extracted the following information from 22 RCTs: the first author, year of publication, study ID, disease stage, trial phase, histology type, treatment regimen, prior treatment history, and the total number of patients treated with ICIs such as PD-1/PD-L1 or CTLA-4 inhibitors. Additionally, they recorded the number of patients with CIP of all grades and grades 3–5.

The quality assessment of the included RCTs was performed using the Cochrane Collaboration’s ’Risk of Bias’ tool [11]. Any disagreements between the two authors (KY and HL) were resolved through consensus, with the involvement of a third senior author (XX-c).

### Statistical analysis

The OR for both overall grade and grade 3–5 CIP was computed employing the inverse variance weighting method among patients administered with PD-1/PD-L1 or CTLA-4 inhibitors (trial group) and those receiving chemotherapy (control group). The calculation included 95% confidence intervals (CIs) and p-values, with the amalgamated analysis executed using the Mantel-Haenszel method. To evaluate heterogeneity, Cochran’s Q test and I2 statistics were utilized. In instances where I2 exceeded 50%, a random-effects model was chosen; otherwise, a fixed-effects model was employed. Subgroup analyses and sensitivity analyses were conducted to probe potential sources of heterogeneity. Additionally, the presence of publication bias in the incorporated studies was assessed through Begg’s and Egger’s tests. Statistical significance was set at a two-sided p-value < 0.05. The software utilized for data analysis was Review Manager 5.3.

## Results

### Literature search results

**Fig 1:**
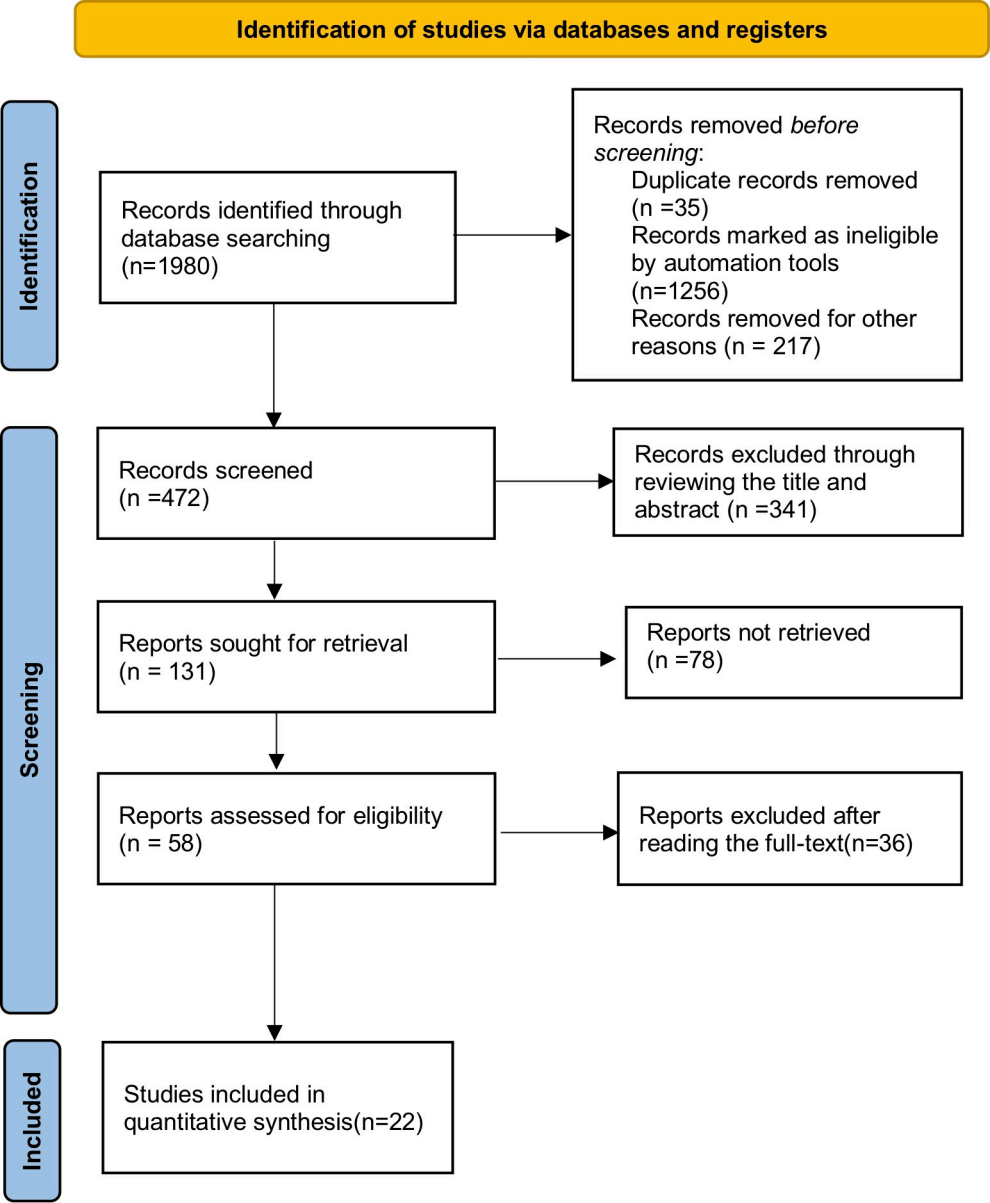
PRISMA flow chart of literature retrieval according to inclusion and exclusion criteria.

In accordance with the search strategy, 1980 articles were initially identified. After screening for adherence to the inclusion and exclusion criteria [7,12–32], 22 randomized controlled trials (RCTs) were ultimately retrieved. Among these, 20 RCTs were extracted from databases such as PubMed, Embase, and Cochrane [7,12–27,29–32], while 2 RCTs were sourced from the American Society of Clinical Oncology (ASCO) and the World Conference on Lung Cancer (WCLC) [28,30]. A comprehensive review of the full texts of these RCTs was conducted, encompassing a total of 13,725 patients diagnosed with NSCLC (Fig 1).

### Study characteristics and quality assessment

Among the 22 included RCTs, 21 were international multicenter phase II-III clinical trials [7,12–31], and one was a multicenter phase III trial (CameL) conducted in 52 Chinese hospitals [32]. Notably, 15 trials involved PD-1 inhibitors (nivolumab, pembrolizumab, cemiplimab, camrelizumab) [12,13,15–18,21–24,26,27,29,31,32], six trials focused on PD-L1 inhibitors (atezolizumab, avelumab) [7,14,20,25,28,30], and one trial examined a CTLA-4 inhibitor (Ipilimumab) [19]. All the RCTs aimed to assess and compare the risk of CIP at all grades and grades 3–5 in patients treated with ICIs versus those treated with chemotherapy controls. Patient stratification was performed across five subgroups based on histology, treatment regimen, PD-L1 expression level, negative EGFR/ALK expression, and previous treatment history.

For non-squamous NSCLC, there were six relevant studies [12,16,21,26,30,32], while four studies focused on squamous NSCLC [13,19,2,28]. Twelve RCTs did not differentiate based on histology. The comparisons included ICI versus single-agent chemotherapy (docetaxel) in eight studies [12–15,20,26,29], ICI versus combination chemotherapy (pemetrexed/carboplatin or paclitaxel/carboplatin) in five studies [15,18,22,29], and ICI combined with chemotherapy versus chemotherapy (pemetrexed/carboplatin or paclitaxel/carboplatin or docetaxel) in nine studies [16,19,21,23,25,27,28,30,32].

For PD-L1 expression, four studies focused on PD-L1 > 1% [15,18,22,29], while three studies investigated PD-L1 > 50% [17,24,31]. In six studies with EGFR/ALK negativity, patients were stratified based on PD-L1 expression into PD-L1 < 1% and PD-L1 > 1% in five studies [16,21,25,30,32], and PD-L1 > 50% in three studies [17,24,31]. The studies were further categorized as previously untreated [7,16–18,22–25,28,31,32] and previously treated [12–15,19–21,26,27,29,30], totaling 11 items (Table 1).

**Table 1:** Characteristics of RCTs included in the meta-analysis.

First author	Year	Study ID	Disease stage	Trial phase	Histology type	Treatment regimen	No. of patients	All grade	Grade 3–5
Borghaei et al. [12]	2015	CheckMate057	IIIB, IV	III	Non-squamous	NIV DOC	287 268	17 23	10 14
Brahmer et al. [13]	2015	CheckMate017	IIIB	III	Squamous	NIV DOC	131 129	6 0	1 0
Fehrenbacher et al. [14]	2016	POPLAR	IIIB, IV	II	Squamous Non-squamous	ATE DOC	144 143	14 4	8 2
Herbst et al. [15]	2016	KEYNOTE‐010	IIIB, IV	II- III	Squamous	PEM DOC	339 309	16 6	7 2
Langer et al. [16]	2016	KEYNOTE-021	IIB	III	Non-squamous	PEM + PBC PBC	59 62	1 0	1 0
Reck et al. [17]	2016	KEYNOTE-024	IV	III	Squamous Non-squamous	PEM PC	154 150	9 1	4 1
Carbone et al. [18]	2017	CheckMate-026	IV	III	Squamous	NIV PC	267 263	7 0	4 0
Govindan et al. [19]	2017	CA184‐104	IV	III	Squamous	IPI +PBC PBC	388 361	8 3	6 2
Rittmeyer et al. [20]	2017	OAK	IIIB, IV	III	Squamous Non-squamous	ATE DOC	609 578	6 0	4 0
Barlesi et al. [7]	2018	JAVELIN Lung200	IIIB, IV	III	Squamous Non-squamous	AVE DOC	393 365	2 14	0 8
Gandhi et al. [21]	2018	KEYNOTE-189	IV	III	Non-squamous	PEM + PBC PBC	404 202	18 5	11 4
Hellmann et al. [22]	2018	CheckMate-227	IV	III	Squamous Non-squamous	IPI + PBC PBC	391 570	9 3	6 2
Paz-Ares et al. [23]	2018	KEYNOTE-407	IV	III	Squamous	PEM +PBC PBC	278 280	18 6	7 3
Mok et al. [24]	2019	KEYNOTE-042	IIIB, IV	III	Squamous Non-squamous	PEM PC	636 615	53 3	22 1
West et al. [25]	2019	IMpower130	IV	III	Non-squamous	ATE + PBC PBC	473 232	18 2	14 1
Wu et al. [26]	2019	CheckMate078	IIIB, IV	III	Squamous Non-squamous	NIV DOC	337 156	10 0	4 0
Arrieta et al. [27]	2020	PROLUNG	IV	II	Squamous Non-squamous	PEM+ DOC DOC	40 38	9 0	2 0
Jotte et al. [28]	2020	IMpower131	IV	III	Squamous	ATE +TC TC	332 334	11 2	9 2
Herbst et al. [29]	2020	KEYNOTE‐010	IIIB, IV	II- III	Squamous	PEM DOC	682 309	40 6	18 2
Nishio et al. [30]	2021	IMpower132	IV	III	Non-squamous	ATE + PBC PBC	291 274	18 6	6 3

**Fig 2:**
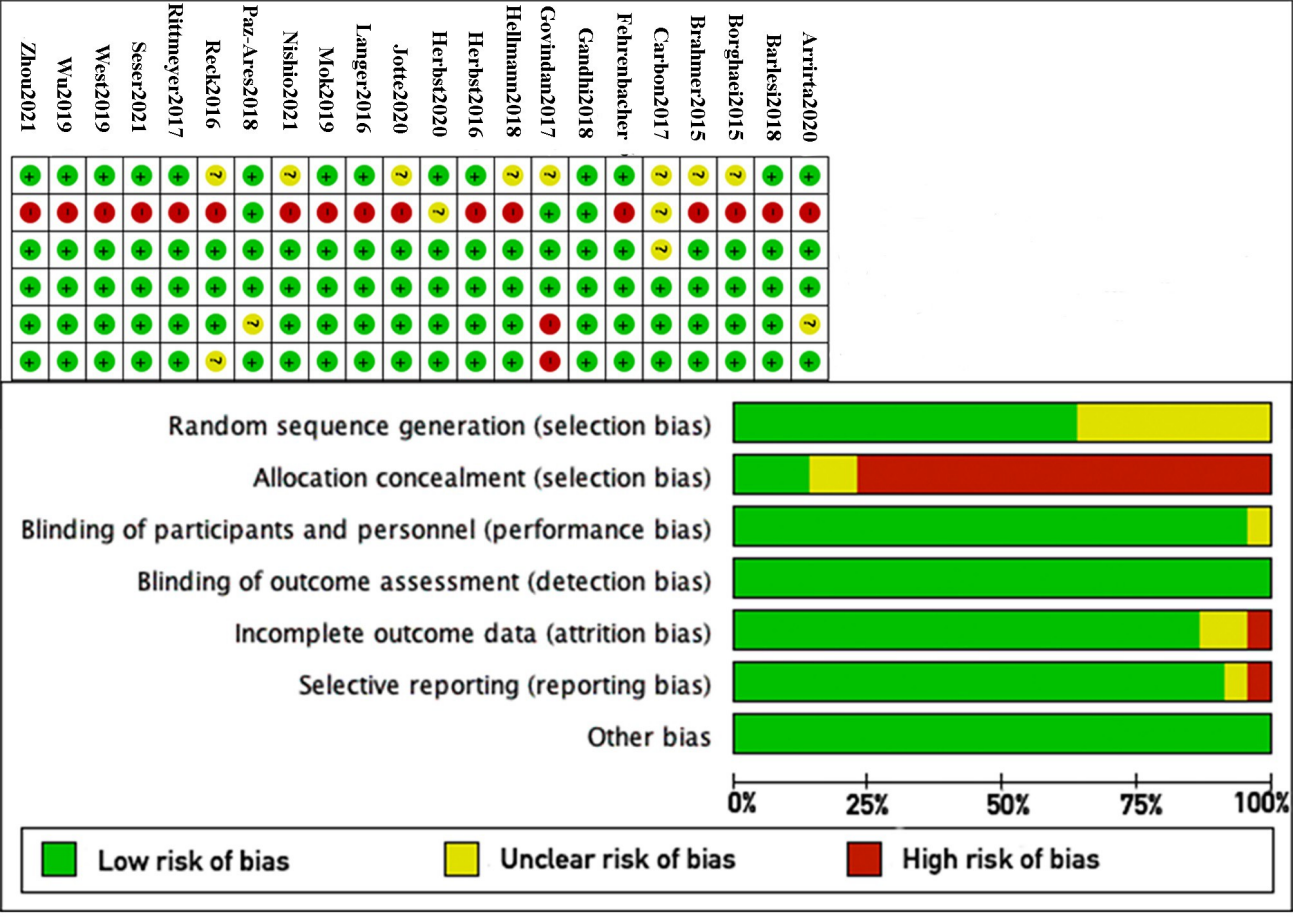
Methodological quality of the included RCTs.

**Fig 3:**
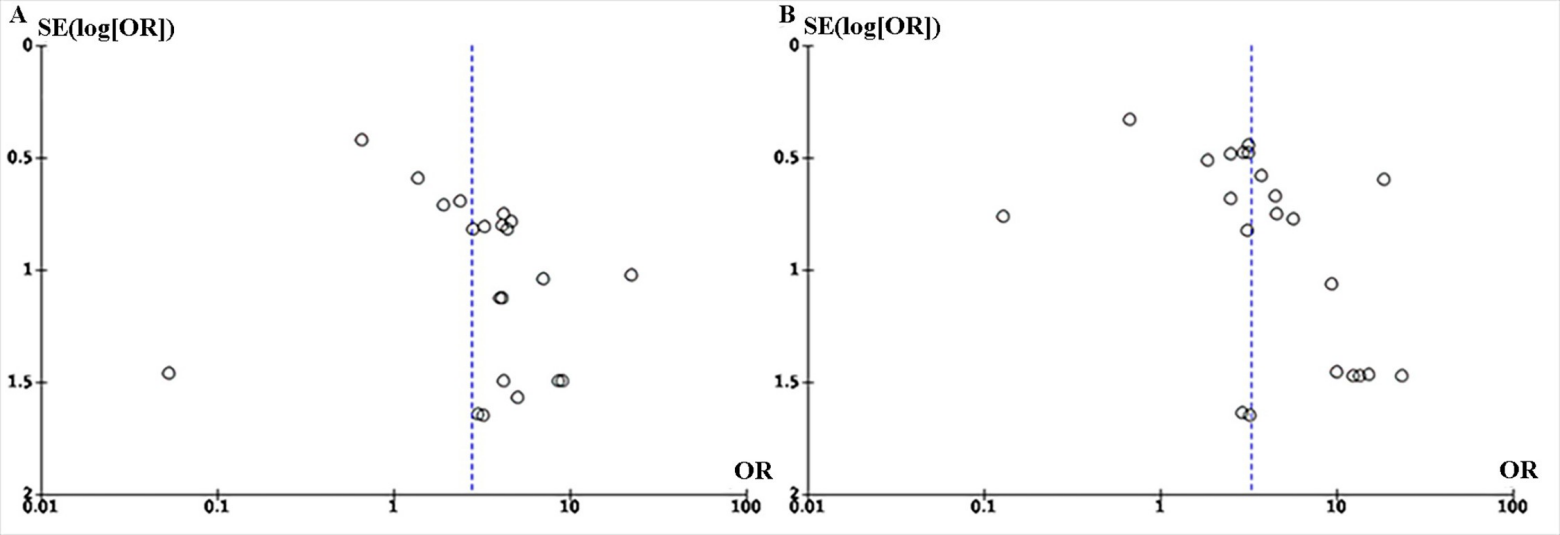
Begg’s funnel plot in evaluation of publication bias. **A**: Funnel plot for Any Grade. **B**: Funnel plot for Grade 3–5.

Most of the 22 RCTs provided details regarding random sequence generation. Among them, 18 utilized open-label randomization [7,12–17,20,22,24–32]. Two RCTs employed a double-blind design [21,23], while the remaining 20 RCTs were either not blinded or exhibited incomplete blinding [7,12–20,22,24–32]. However, the authors asserted that the lack of blinding did not impact the study outcomes. Only one RCT failed to reach the study endpoint [19], potentially compromising the integrity of the outcome data. All RCTs were deemed to be at low risk of measurement bias, and no other biases were identified (Fig 2). There was no evidence of publication bias (Fig 3).

### Risk and subgroup analysis of CIP of all grade and 3–5 grade in advanced NSCLC Histology

In the EGFR and ALK wild-type non-squamous subgroup, ICI increased the risk of CIP of all grades compared to chemotherapy (OR = 2.72, 95% CI 1.54–4.82, p = 0.0006). The risk of grade 3–5 CIP with ICI was slightly higher (OR = 2.23, 95% CI 1.06–4.69, p = 0.03). In the subgroup of non-squamous with unknown EGFR and ALK status, the comparison between ICI and chemotherapy showed no significant difference in the risk of CIP for all grades and grades 3–5 (OR = 0.67, 95% CI 0.35–1.29, p = 0.23; OR = 0.65, 95% CI 0.29–1.50, p = 0.32).

**Fig 4:**
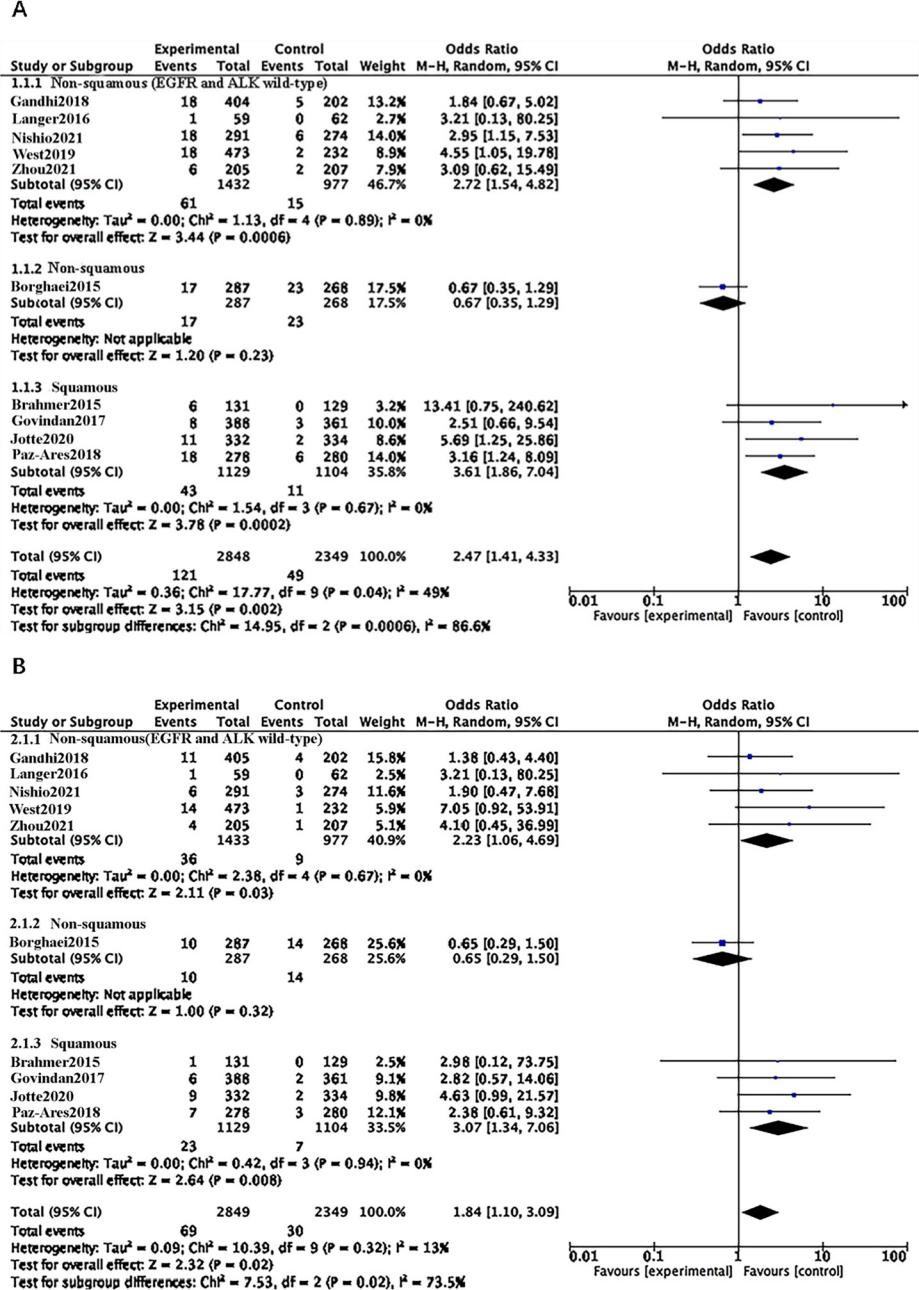
**A:** Forest plots of RRs comparing Any Grade between ICI and chemotherapy in the different histology subgroup. **B:** Forest plots of RRs comparing Grade3-5 between ICI and chemotherapy in the different histology subgroup.

Within the squamous subgroup, ICI also increased the risk of CIP for all grades and grade 3–5 compared to chemotherapy (OR = 3.61, 95% CI 1.86–7.04, p = 0.0002; OR = 3.07, 95% CI 1.34–7.06, p = 0.008). Among the three subgroups, the squamous subgroup exhibited a higher risk of developing CIP for all grades and grades 3–5 (OR = 3.61, 95% CI 1.86–7.04, p = 0.0002; OR = 3.07, 95% CI 1.34–7.06, p = 0.008). In a post-combination analysis of the three subgroups, ICI increased the risk of all-grade and grade 3–5 CIP compared to chemotherapy (OR = 2.47, 95% CI 1.41–4.33, p = 0.002; OR = 1.84, 95% CI 1.10–3.09, p = 0.02) (Fig 4).

### Treatment regimen

They were divided into three subgroups based on the treatment plan: ICI vs. Single-agent CT, ICI vs. Combination-agent CT, and ICI+CT vs. CT. In the ICI vs. Single-agent CT subgroup, the risk of developing CIP of all grades between ICI and single-agent CT was not significantly different (OR = 2.02, 95% CI 0.80–5.09, p = 0.14), whereas the risk of grade 3–5 CIP with ICI was slightly higher (OR = 2.37, 95% CI 1.05–5.33, p = 0.04). There was moderate heterogeneity in the ICI vs. Single-agent CT subgroup (I^2^ = 76%).

**Fig 5:**
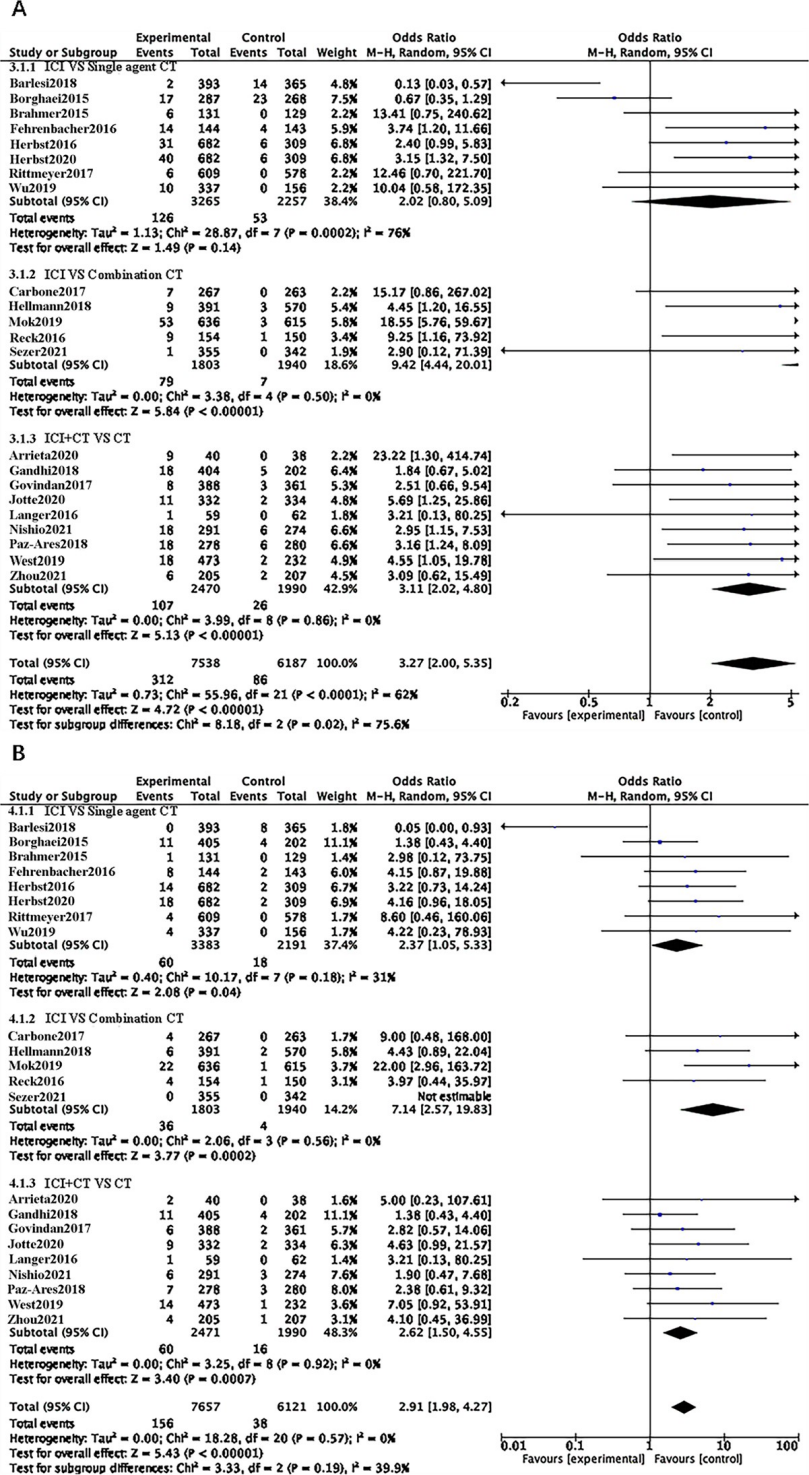
**A:** Forest plots of RRs comparing Any Grade between ICI and chemotherapy in the different treatment regimen subgroup. **B:** Forest plots of RRs comparing Grade3-5 between ICI and chemotherapy in the different treatment regimen subgroup.

In the two subgroups of ICI versus Combination Agent CT and ICI+CT versus CT, both ICI monotherapy and combination chemotherapy increased the risk of CIP of all grades and grades 3–5 compared with chemotherapy. For the ICI versus Combination Agent CT subgroup, the risk of developing all grades of CIP was OR = 9.42, 95% CI 4.44–20.01, p<0.00001; while the risk of developing grade 3–5 CIP was OR = 7.14, 95% CI 2.57–19.83, p = 0.0002. For the ICI+CT versus CT subgroup, the risk of developing all grades of CIP was OR = 3.11, 95% CI 2.02–4.80, p < 0.00001; while the risk of developing grades 3–5 CIP was OR = 2.62, 95% CI 1.50–4.55, p = 0.0007. After a combined analysis of the effect sizes of the three subgroups, ICI, regardless of being used as a single agent or in combination with chemotherapy, presented a higher risk of CIP of all grades and grades 3–5 than single-agent or combination chemotherapy alone (OR = 3.27, 95% CI 2.00–5.35, p <0.00001; OR = 2.91, 95% CI 1.98–4.27, p<0.00001) (Fig 5).

### PD-L1 expression level

**Fig 6:**
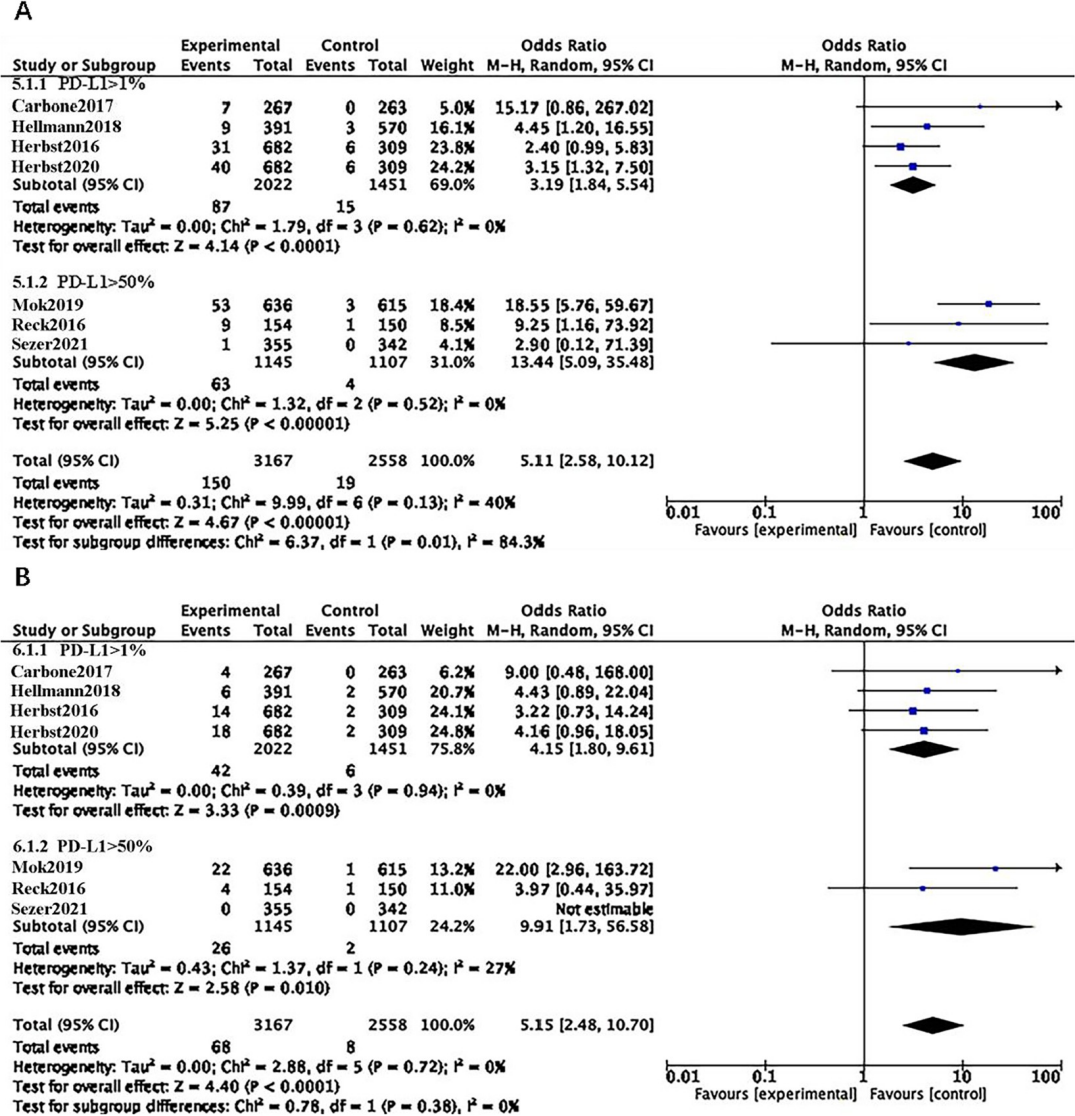
**A:** Forest plots of RRs comparing Any Grade between ICI and chemotherapy in the different PD-L1 expression level subgroup. **B:** Forest plots of RRs comparing Grade3-5 between ICI and chemotherapy in the different PD-L1 expression level subgroup.

We divided the study population into two subgroups based on PD-L1 expression levels: PD-L1 > 1% and PD-L1 > 50%. Our findings indicate that, irrespective of whether PD-L1 expression was low or high, ICI treatment led to an increase in adverse events of all grades and grades 3–5, compared with chemotherapy (OR = 5.11, 95% CI 2.58–10.12, p < 0.00001; OR = 5.15, 95% CI 2.48–10.70, p < 0.0001). Subgroup analysis revealed that the PD-L1 > 50% group faced a higher risk of developing immune-related adverse events of all grades and grades 3–5 (OR = 13.44, 95% CI 5.09–35.48, p < 0.00001; OR = 9.91, 95% CI 1.73–56.58, p = 0.01). In patients with positive PD-L1 expression, although they benefited from ICI therapy, there was also an increased risk of immune-related adverse events (Fig 6).

### EGFR/ALK-negative

**Fig 7:**
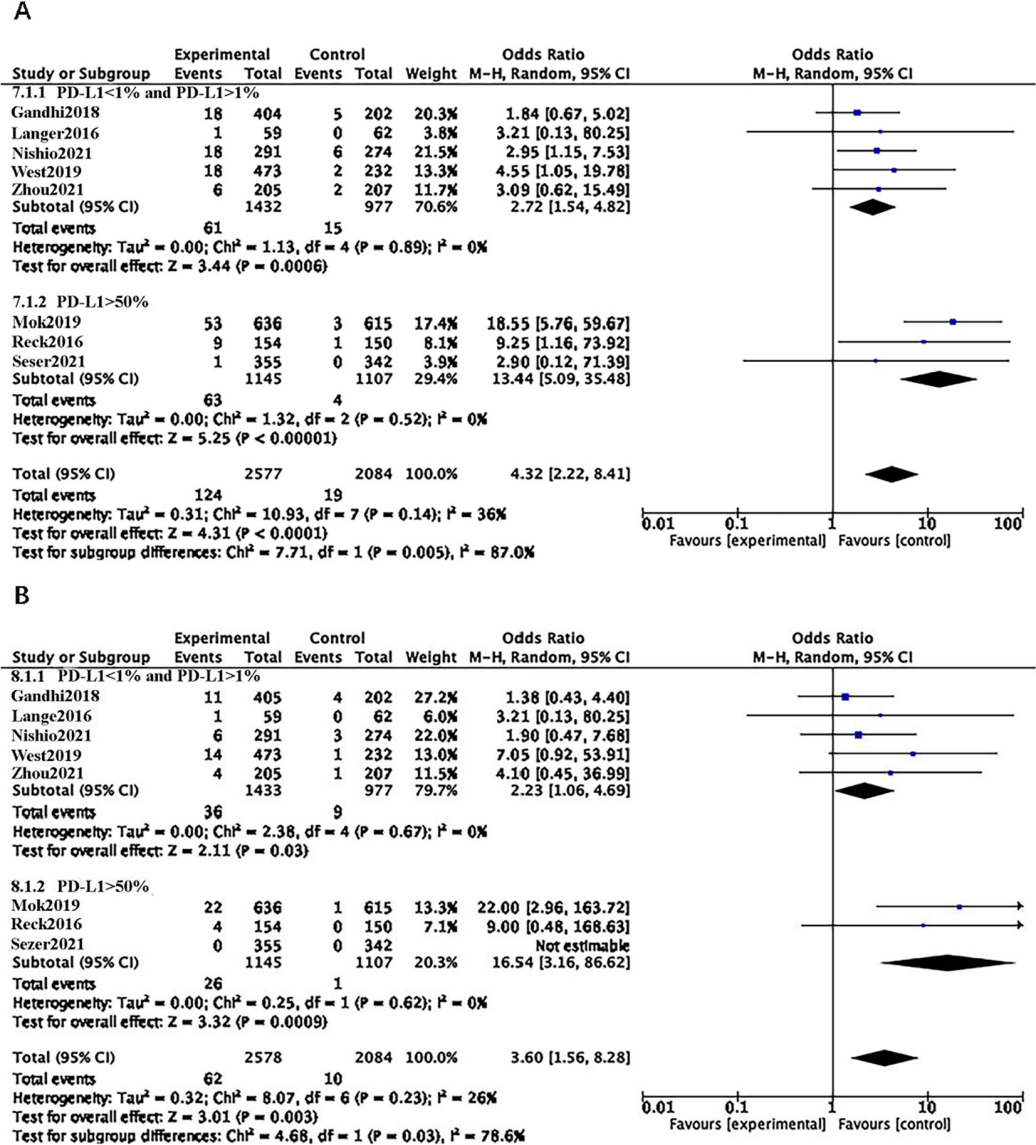
**A**: Forest plots of RRs comparing Any Grade between ICI and chemotherapy in the EGFR/ALK-negative subgroup. **B**: Forest plots of RRs comparing Grade 3–5 between ICI and chemotherapy in the EGFR/ALK-negative subgroup.

Among the seven RCTs involving patients with negative EGFR/ALK driver genes, four encompassed individuals with both negative and low PD-L1 expression (PD-L1 < 1% and PD-L1 > 1%), while the remaining three RCTs focused on patients exhibiting high PD-L1 expression (PD-L1 > 50%). The findings revealed that ICI increased the risk of CIP compared to chemotherapy, irrespective of PD-L1 expression levels (OR = 4.32, 95% CI: 2.22–8.41, p < 0.0001; OR = 3.60, 95% CI: 1.56–8.28, p = 0.003). Subgroup analysis further indicated that the group with PD-L1 > 50% had a higher risk of developing CIP of all grades and grades 3–5 (OR = 13.44, 95% CI: 5.09–35.48, p < 0.00001; OR = 9.91, 95% CI: 1.73–56.58, p = 0.01) (Fig 7).

### History of previous treatment

**Fig 8:**
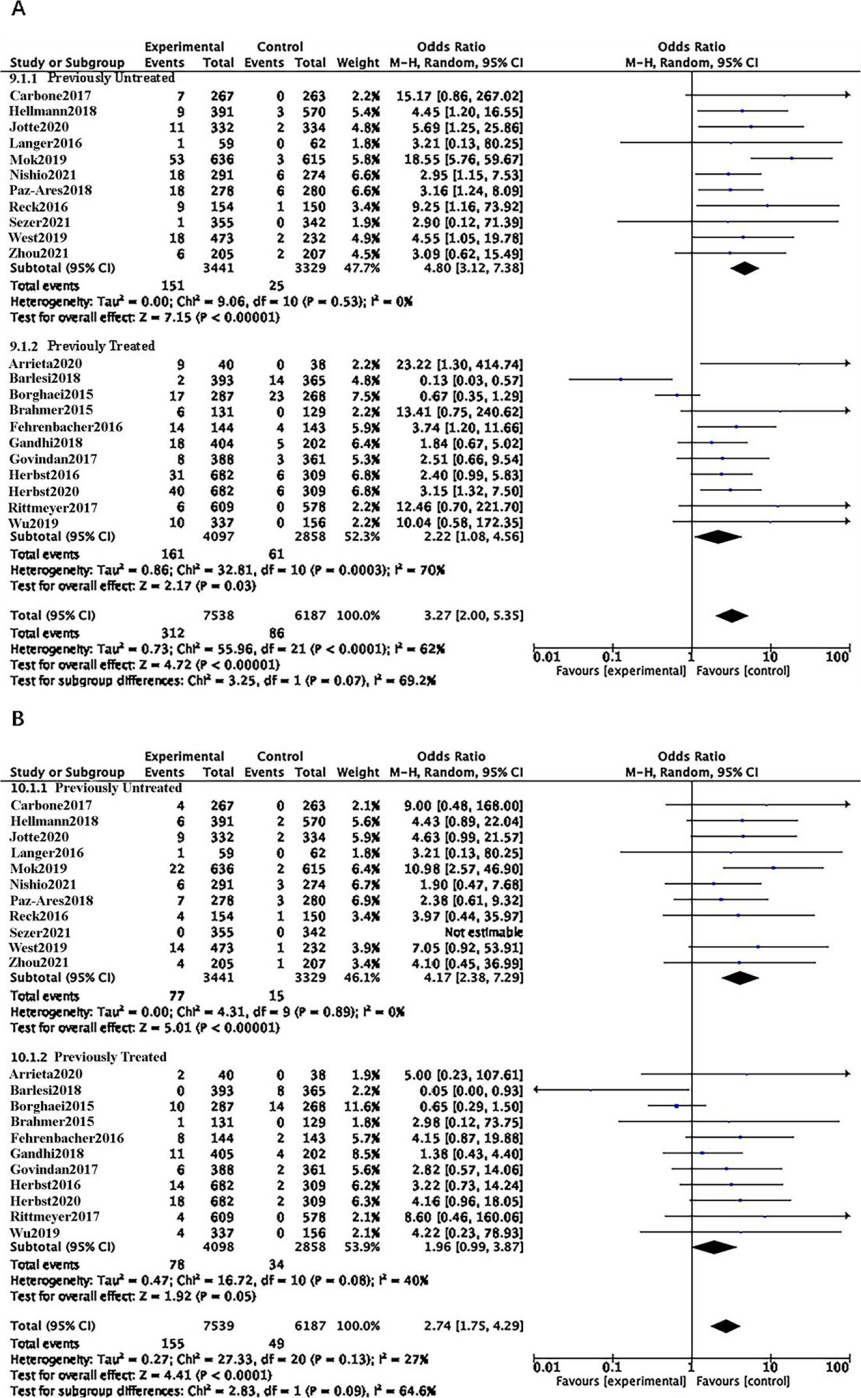
**A**: Forest plots of RRs comparing Any Grade between ICI and chemotherapy in the previous treatment history subgroup. **B**: Forest plots of RRs comparing Grade 3–5 between ICI and chemotherapy in the previous treatment history subgroup.

In the previously untreated subgroup, ICI increased the risk of all-grade and grades 3–5 CIP compared with chemotherapy (OR = 4.80, 95% CI 3.12–7.38, p<0.00001; OR = 4.17, 95% CI 2.38–7.29, p<0.00001). In the previously treated subgroup, ICI slightly elevated the risk of all-grade CIP compared with chemotherapy (OR = 2.22, 95% CI 1.08–4.56, p = 0.03), while the risk of grade 3–5 CIP was not significantly different (OR = 1.96, 95% CI 0.99–3.87, p = 0.05). Combining the effect sizes of the two subgroups revealed that ICI increased the risk of all-grade and grade 3–5 CIP compared with chemotherapy, irrespective of prior treatment history (OR = 3.27, 95% CI 2.00–5.35, p<0.00001; OR = 2.74, 95% CI 1.75–4.29, p<0.0001). Moderate heterogeneity was observed in the previously treated subgroup (I^2^ = 70%, I^2^ = 40%), and sensitivity analysis identified JAVELIN Lung200 and CheckMate057 as sources of the heterogeneity (Fig 8).

## Discussion

CIP is a severe and potentially life-threatening adverse event. CIP is characterized by dyspnea or other respiratory symptoms, such as cough and post-activity shortness of breath, alongside new infiltrates on chest imaging after ICI therapy, excluding clinically new pulmonary infection or tumor progression [5]. In our analysis, we incorporated data from 22 RCTs that compared the risk of CIP associated with PD-1/PD-L1 or CTLA-4 inhibitor use alone or in combination with chemotherapy. Our meta-analysis revealed that in advanced NSCLC, irrespective of histological subtype (non-squamous or squamous), presence or absence of chemotherapy, high or low PD-L1 expression levels, and prior treatment history, the incidence of CIP was higher with ICI compared to chemotherapy for all grades and grades 3–5 adverse events in advanced NSCLC patients. Subgroup analysis further demonstrated that patients with squamous histology, high PD-L1 expression, and no prior treatment history were more susceptible to developing CIP of all grades and grades 3–5. This study represents the first comprehensive analysis and comparison of CIP risk in advanced NSCLC with varying histologies, treatment regimens, PD-L1 expression levels, and treatment histories. The insights gained from these findings may assist clinicians in enhancing the management of pulmonary toxicity associated with immunotherapy in advanced lung cancer patients.

We discovered that both the EGFR and ALK wild-type non-squamous subgroups, as well as the squamous subgroups, exhibited a higher risk of CIP in the ICI group compared to chemotherapy, irrespective of the grade (all grades or grade 3–5). Within the non-squamous subgroup, there was no significant difference in the risk of developing CIP for all grades and grade 3–5. The RCT for this subgroup was CheckMate 057 [12], which included patients with EGFR mutations or ALK translocations. Among these patients, those with EGFR mutations and ALK translocations comprised 19% in the ICI (nivolumab) group and 16% in the chemotherapy (docetaxel) group. Although immunotherapy has been applied to treat patients with driver gene mutations, it is generally not recommended unless the patient’s disease has progressed after treatment with the latest generation of targeted drugs. For patients with EGFR gene mutations, particular caution should be exercised when using PD-1/PD-L1 inhibitors either before or concurrently with EGFR-TKIs, as there have been reports indicating an increased risk of pulmonary toxicity when these drugs are used in combination [33]. Regarding the histological type of NSCLC, previous studies have indicated that patients with squamous NSCLC have a higher incidence of CIP than those with non-squamous NSCLC [34,35]. Additionally, some studies suggest a higher incidence of CIP in lung adenocarcinoma [36]. Our findings further support the notion that patients with squamous NSCLC are at a higher risk of developing CIP for all grades and grades 3–5.

In our study, we did not observe a statistical difference in the risk of CIP between ICI and single-agent CT with docetaxel. However, the ICI group exhibited a higher risk of CIP compared to the combination of CT and ICI versus CT alone. The factors contributing to CIP risk remain elusive. Variables such as gender, advanced age, smoking history, decreased basal pulmonary function, and a history of lung surgery or radiotherapy may be associated with the occurrence of CIP [37–39]. Nevertheless, substantial evidence supporting these correlations is still lacking. It is noteworthy that patients in the ICI versus single-agent CT subgroup had poorer baseline lung function and were unable to tolerate double-agent chemotherapy, resulting in increased CIP intolerance. Notably, there was significant heterogeneity in the ICI group compared to the single-agent chemotherapy group (I^2^ = 76%). Further analysis revealed that this heterogeneity stemmed from the JAVELIN Lung200 and CheckMate057 studies. Upon excluding these studies, the heterogeneity reduced to 0. In the JAVELIN Lung200 study, the ICI used was a PD-L1 inhibitor, while other studies employed PD-1 inhibitors. A meta-analysis of 23 randomized controlled trials (RCTs) indicated a relatively low incidence of all-grades and grades 3–5 CIP (3.25%, 2.12%). Moreover, there was no significant difference in CIP incidence between various PD-1 inhibitors, and CTLA-4 inhibitors did not appear to elevate the risk of CIP [7]. In the CheckMate057 study, ECOG scores were not balanced between the two groups, and there were more patients over 75 years old in the single-agent chemotherapy group (docetaxel). Cho et al. reported that patients with CIP are typically over 70 years old [40]; however, conflicting studies suggest that advanced age does not necessarily increase the risk of CIP with ICI [41]. The impact of age on the risk of developing CIP with immunotherapy has not been systematically studied.

The expression level of PD-L1 stands out as a crucial factor when determining initial treatment options for advanced NSCLC. The KEYNOTE-024 study revealed that the PD-1 inhibitor pembrolizumab surpassed standard chemotherapy for NSCLC patients exhibiting a PD-L1 Tumor Proportion Score (TPS) of 50% [17]. For individuals with advanced NSCLC lacking driver gene mutations and PD-L1 expression greater than 1%, particularly those with PD-L1 expression exceeding 50%, the recommendation is to consider PD-1/PD-L1 inhibitor monotherapy or combination chemotherapy. Wang et al. conducted a systematic review and network meta-analysis (NMA), demonstrating that the combination of chemotherapy with immunotherapy significantly correlated with an enhanced Overall Response Rate (ORR) and Progression-Free Survival (PFS) when compared to immune checkpoint inhibitors alone. However, there was not a substantial improvement in Overall Survival (OS) [42]. Given the inclusion of randomized controlled trials, it’s essential to note that this analysis lacks the statistical power to replace direct head-to-head clinical trial comparisons. Consequently, the optimal first-line treatment, either chemotherapy combined with immunotherapy or ICI monotherapy, for patients with advanced NSCLC and high PD-L1 expression remains a subject of debate [43]. Our study identified that, irrespective of the PD-L1 expression level, ICI carried a higher risk of all-grades and grades 3–5 CIP compared to chemotherapy, particularly in patients with elevated PD-L1 expression. In driver-negative advanced NSCLC, within the subgroups of PD-L1<1% and PD-L1>1%, ICI presented a slightly higher risk of grade 3–5 adverse effects than chemotherapy. The inclusion of PD-L1-negative patients in this scenario remains unclear. A retrospective cohort study discovered an association between grade 1–2 pneumonitis and ICI efficacy in NSCLC [41]. The predictive value of CIP occurrence in NSCLC patients undergoing PD-1/PD-L1 inhibitor therapy remains uncertain. Suresh et al. found increased lymphocytes (mainly CD4 + T cells) and decreased PD-1 and CTLA-4 expression in regulatory T cell populations in bronchoalveolar lavage fluid (BALF) samples from CIP patients [44]. Future studies need more evidence to explore the mechanism of CIP.

Previous studies have indicated a potential association between the occurrence of CIP and the use of multiple lines of therapy. The Keynote-001 trial, for instance, identified a heightened incidence of CIP of all grades among patients treated with a PD-1 inhibitor (pembrolizumab) who had previously undergone radiotherapy, as compared to those who hadn’t (13% vs. 1%, p < 0.05) [45]. Another study has suggested that the timing, duration of treatment, and dose of thoracic radiotherapy are not correlated with the occurrence of CIP [46]. However, it is essential to note that radiotherapy itself can lead to dose-dependent and volume-dependent radiation pneumonitis. Unlike CIP, most lesions resulting from radiotherapy are confined to the radiation area. Some patients may exhibit no apparent respiratory symptoms, and bronchoalveolar lavage often reveals an increased proportion of lymphocytes. In a meta-analysis conducted by Khunger et al., a higher incidence of grade 1–4 pneumonitis was observed in untreated patients compared to those who had undergone previous treatment (4.3% vs. 2.8%, p = 0.03) [47]. Our study aligns with these findings, revealing that untreated patients face a higher risk of developing CIP of all grades or grades 3–5 than those with a history of treatment. Additionally, patients treated with Immune Checkpoint Inhibitors (ICI) following prior treatment showed a slightly elevated risk of CIP of all grades compared to those undergoing chemotherapy. However, the difference in grades 3–5 CIP was not statistically significant. Notably, we observed moderate heterogeneity (I^2^ = 70% for all grades, I^2^ = 40% for grades 3–5) within the subgroup of patients with a history of prior treatment. Similar heterogeneity was also noted in JAVELIN Lung200 and CheckMate057.

The era of NSCLC immunotherapy appears to have arrived. However, patients benefiting from immunotherapy still lack validated biomarkers of response, such as programmed death ligand 1 (PD-L1) expression, tumor mutation burden (TMB), microsatellite instability (MSI) status, and intestinal microbiota. The immunomodulatory effects produced by drugs like PPIs may impair the activity of ICIs, thereby altering the intestinal microbiota. A meta-analysis has suggested that PPIs and H2RAs may impact the efficacy of ICIs in patients with metastatic NSCLC receiving immunotherapy. Due to the relatively small and underpowered nature of most included studies, more extensive prospective clinical trials are necessary for verification [48]. The MOUSEION-01 study delved into the influence of gender on the efficacy of immune checkpoint inhibitors in cancer patients. The primary endpoint aimed to assess overall survival (OS) in male and female patients who received immune checkpoint inhibitors versus control therapy. In the lung cancer subgroup analysis, it was observed that women experienced greater benefits [49]. In the MOUSEION-03 study, a systematic evaluation was conducted to explore the possibility of achieving complete response (CR) in cancer patients receiving ICIs. The results indicated that, compared with control treatment, patients undergoing immunotherapy and chemotherapy combined with immunotherapy exhibited higher CR rates, particularly in those with metastatic non-small cell lung cancer [50]. However, the underlying mechanism of CR still requires further research.

Our current meta-analysis, which reports on the risk of CIP in advanced NSCLC, has several limitations. Firstly, the majority of the included RCTs were open-label, potentially introducing allocation concealment selection bias. Secondly, our meta-analysis primarily focused on PD-1/PD-L1 inhibitors as the main immunotherapy agents, with only one study addressing CTLA-4 inhibitors due to a lack of published data at the time of data collection for this particular study. Thirdly, CIP lacks typical clinical symptoms and imaging manifestations, and there exists no standardized diagnostic criteria and process. Currently, CIP remains a diagnosis of exclusion, requiring the exclusion of infections and malignant tumor progression. Consequently, the identification of CIP in the included RCTs may not be entirely accurate.

## Conclusion

CIP represents a notably severe adverse reaction associated with advanced lung cancer immunotherapy, exhibiting a risk profile influenced by histology, treatment regimen, PD-L1 expression level, and previous treatment history. Our study discerned that, in advanced NSCLC, ICI posed a heightened risk of CIP compared to chemotherapy. Conversely, patients with squamous histology, elevated PD-L1 expression, and an absence of prior treatment history demonstrated a greater likelihood of developing CIP. Despite these findings, the comprehensive elucidation of CIP risk factors remains incomplete. There is a pressing need for further research into the histology and biological characteristics of CIP, coupled with extensive exploration of risk stratification. This will facilitate a more nuanced understanding, enabling clinicians to fortify their approach to managing immunotherapy-related lung diseases. This, in turn, will pave the way for more precise and tailored therapeutic interventions for specific patient subgroups.

## Supporting information

S1 Checklist PRISMA 2020 checklist. (DOCX)

S1 Data (XLSX)
